# Geographic availability and accessibility of day care services for people with dementia in Ireland

**DOI:** 10.1186/s12913-020-05341-z

**Published:** 2020-05-27

**Authors:** Tom Pierse, Fiona Keogh, Eamon O’Shea, John Cullinan

**Affiliations:** 1 Centre for Economic and Social Research on Dementia, National University of Ireland Galway, Galway, Ireland; 2 School of Business & Economics, National University of Ireland Galway, Galway, Ireland

## Abstract

**Background:**

Day care is an important service for many people with dementia and their carers. In Ireland, day care services for people with dementia are delivered by a mix of dementia-specific day care centres as well as generic day care centres that cater for people with dementia to various degrees. In this paper we examine the geographic distribution of day care services for people with dementia relative to potential need.

**Methods:**

Using a national survey of day care centres, we estimate the current availability of day care services for people with dementia in the country. We use geographic information systems (GIS) to map day care provision at regional and sub-regional levels and compare this to the estimated number of people with dementia in local areas.

**Results:**

There is significant variation across the country in the existing capacity of day care centres to cater for people with dementia. The number of places per 100 persons with dementia in the community varies from 14.2 to 21.3 across Community Health Organisation areas. We also show that 18% of people with dementia do not live within 15kms of their nearest day care centre.

**Conclusion:**

Currently, day care centres, in many parts of the country, have limited capacity to provide a service for people with dementia who live in their catchment area. As the number of people with dementia increases, investment in day care centres should be targeted to areas where need is greatest. Our GIS approach provides valuable evidence that can help inform decisions on future resource allocation and service provision in relation to day care.

## Background

Day care services are an important component of community care for people with dementia, with between 10 and 18% of people with dementia in the community utilising the service internationally [1]. In Ireland, an estimated 13% of community care expenditure on dementia goes towards day care [2], the second largest area of community expenditure for people with dementia after home care. Overall there are an estimated 55,000 people with dementia in Ireland, of whom 35,000 live in the community [3]. The number of cases is expanding due to population aging, with current projections indicating that by 2031 there will be 95,863 people living with dementia in Ireland [4]. Therefore, a significant expansion in day care services that cater for people with dementia will be required to maintain, or expand on, the current level of service.

An ‘older persons day care centre’ is a term used to describe a building-based service that offers programmes and services for older people with a variety of needs. Day care for older people can be defined in different ways; a ‘social model’ where centres aim to provide socialisation and activities and a ‘medical model’ where health services and rehabilitation are provided [5]. Overall, people with dementia who attend day care services typically express high degrees of satisfaction [6–8]. Attending a day care centre provides the opportunity for social interaction and a sense of structure and routine [9, 10], and day care has been shown to provide people with dementia with a range of benefits. These include: increased wellbeing [7, 11]; better sleeping habits [11, 12]; reduced neuropsychiatric symptoms and use of psychotropic drugs [12, 13]; and, reduced family carer stress [14, 15]. Having access to day care can also improve the relationship between the carer and the person with dementia by providing time apart and facilitating employment [5].

Demand for day care services for people with dementia is influenced by a number of factors, particularly personal preferences and the appropriateness of the provision to the person’s needs. People with dementia are often reluctant to go to a day care centre, which poses a dilemma for care givers [16, 17]. Many will only seek day care late in the condition when cognition and function have declined significantly [18]. In addition, there are also significant drop out rates among people with dementia with severe behavioural problems and depression [19]. Centres that cater for people with dementia may also have restrictions on enrolment; incontinence and disruptive behaviour are cited as the most common restrictions in the US [20]. Not surprisingly, therefore, day care utilisation rates among people with dementia tend to be low [1].

Similar to other countries, day care for people with dementia in Ireland is delivered though a mix of dementia-specific centres and older persons centres which typically provide for a small number of people with dementia as part of a generic service [20, 21]. Because of this, and limitations in data collection infrastructure in day care centres, a key challenge is in identifying the current provision of day care services for people with dementia in the country. The national survey data reported here, the first survey for Ireland combining national data on location and attendees, takes on a particular significance in this regard. Furthermore, while tools for assessing geographic accessibility have been developed for a range of health services [22], little is known about the geographic accessibility (distance to a day care centre) or availability (supply of places) of day care services for people with dementia. This paper uses geographic information systems (GIS) methods to provide evidence for policy and service planning decisions relating to the allocation of resources in the day care sector, though the approach could be equally applied to other services and other jurisdictions. We take a multi-level approach to the resource allocation problem of identifying the parts of the country with the greatest need. To assist in resource allocation at a national level, we examine disparities across administrative health regions (Community Health Organisations, CHOs) in the availability of day care for people with dementia. We then look at availability within each of these regions to identify sub regions (Community Health Networks, CHNs) with the lowest availability. Finally, we look at access and population density to assist local decision makers in identifying preferred locations for future investment in day care infrastructure within each CHN.

## Methods

### Day care Centre survey

Data on day care centres are sourced from a national survey of Irish day care centres carried out by the Health Service Executive (Ireland’s national health service) in 2018. This survey defined a day care centre as follows:

*“Day Care Centres are open for a minimum of five hours per day for at least one day per week. All clients are referred to the service by healthcare professional e.g. PHN or Primary Care Team Member, usually requiring completion of referral form. Some dementia-specific centres may accept a referral from appropriate health professional or family. Attendance at the Day Care Centre service is open-ended and usually long term”.*

This definition positions day care centres between social activities, such as active retirement groups, which are accessed for fewer hours, and day hospitals where attendance is short term with a specific assessment, treatment or rehabilitative objective.

The Health Service Executive had a list of day care centres which it funds; this provided the main population frame for the national survey. Services operated by the Alzheimer Society of Ireland (ASI) or Western Alzheimer were identified by cross referencing with the ASI/National Dementia Office audit of dementia-specific services [23]. The survey also incorporated data from a similar regional (Cork and Kerry) survey of 41 centres carried out in May 2016. The survey questionnaire was initially piloted by a number of day care centres and amended based on feedback from the pilot. The final survey questionnaire profiled day care centre service activity for 1 week between April 30th 2018 to May 6th 2018 and included questions on the total number of places and actual attendances on each day of that week. The key respondent was the administrator/manager of the day care centre. The survey included questions on the number of places for people with dementia on each day. In addition, the survey gathered information on characteristics of clients, including age category, gender, dementia status and dependency. The dementia status of clients, where available, was based on the report of the respondent and not necessarily based on a validated diagnosis of dementia. Dependency status was predominantly based on a Barthel Index assessment [24]. A comment box allowed respondents to specify the policy in relation to people with dementia attending the day service. The characteristics of the building in which the centre is located and the organisation and funding of the service were also covered in the survey.

A total of 317 day care centres for older people were identified, all of whom responded to the survey. The lack of data collection infrastructure in many of the centres meant that the survey placed a significant demand on personnel running the centre who have little expertise in data collection. However, significant effort and persistence on the part of the survey team, such as through follow up calls, resulted in the 100% response rate. A number of questions in the survey had incomplete or missing data. This was particularly the case in completing client data on age and physical dependency and reflects the generally poor infrastructure for data collection in day centres.

### Identifying categories of day care provision

In order to identify day care places for people with dementia, data from the survey was used to categorise day care centres into four mutually exclusive groups, namely:

1. Dementia-specific day care centre;
2. Dementia-specific days within generic centre;
3. Dementia within generic day care centre;
4. Centre with no dementia activity recorded.

First, a centre was categorised as a “dementia-specific” centre where: i) the centre was operated by the Alzheimer Society of Ireland (ASI) or the Western Alzheimer Society (collectively referred to as ‘Alzheimer Societies’ throughout); ii) qualitative comments in the survey indicated that the centre was dementia-specific; iii) over 4 days per week were dementia-specific; iv) all clients were identified as having dementia. A small number of dementia-specific centres were co-located with generic day centres for older people. These centres are typically run by the Alzheimer Society of Ireland for one or 2 days a week, with the generic centre operating on other days. In these cases, where two surveys were returned, the centres were recorded as two separate entities – one a dementia-specific centre, and the second a generic day centre.

In the second category, a centre was designated as “dementia-specific days” where: i) the generic centre specified certain days where all places were dedicated for people with dementia and no other people attended, while on other days there was a mix of people with and without dementia; ii) qualitative comments in the survey indicated that the centre operated dementia-specific days. The third category occurred when people with dementia attended generic day care services, attending alongside other users, but not generally receiving dementia-specific attention. Finally, centres were categorised as “no dementia recorded” where: i) qualitative comments reported that the centre did not take people with dementia or that the centre did not have any people with dementia attending; ii) there were no records of dementia-specific days, dementia places or attendees with dementia. While these centres may not have had an explicit policy of not accepting people with dementia, there was no indication from the data that they did cater for people with dementia.

### Assessing the capacity of day care centres to cater for people with dementia

The aim of this study is to identify geographic variation in the provision of day care for people with dementia to assist in the planning of community services for people with dementia. We generate an estimate of the number of weekly day care places that are available to people with dementia, irrespective of whether these are provided in a dementia-specific centre or a generic centre for older people. We examine capacity on a weekly basis taking into account the variation in the number of days that centres open; some centres open 5 days a week while others only open for 1 day a week. Therefore, the weekly number of places is the best comparator of service availability. For example, a centre with ten places that runs for 3 days a week is deemed to have 30 weekly places.

Due to incomplete data in the surveys, the capacity of centres to cater for people with dementia is not directly evident. To generate an estimate of the number of people with dementia that a centre caters for, a set of rules were used to compile responses to the relevant questions in the survey. A separate approach to estimating the number of weekly places for people with dementia is adopted for each category of day care centre. For dementia-specific centres, all weekly places are deemed to be dementia places. While this is accurate for Alzheimer Society centres that require a diagnosis for people to use the services, the approach may overestimate the number of dementia places available in a small number of other centres, as they may not require a diagnosis to use the service. For centres categorised as having dementia-specific days, the number of places available on dementia specific days is used as the estimate of the number of dementia places.

For centres that cater for people with dementia within a general service, we sought to identify the proportion of the service being used by people with dementia. This was identified from the data on the dementia status of individual attendees. While there was a significant level of incomplete data in this variable, it provides a basis for estimating the extent to which the service was directed towards people with dementia. This approach was augmented with qualitative comments in the survey that identified the number of people with dementia and the number of places specifically designated for people with dementia in the generic service.

### Spatial analysis

#### Geographic areas

We examine the availability of day care for people with dementia at two different geographic levels. First, there are nine CHO areas in Ireland with an average population of 529,000 people. This is an important administrative area for community health resource allocation decisions. The second level we examine are CHN areas, of which there are 96, and there are typically around 10 CHNs in a CHO. These areas have been recently delineated and are likely to become increasingly important in the allocation of community resources in the coming years. The estimated number of people with dementia living in the community in CHN areas ranges from 104 to 700 across Ireland, based on estimates for 2016 [3].

To examine accessibility, we use a smaller geographical area – electoral district (ED) areas – to estimate distances from day care centres to the population with dementia. There are 3441 EDs in Ireland, which have an average population of 1397 and cover an average area of 20 km^2^. All spatial and data analyses were performed within a GIS environment (ESRI® ArcGIS® ArcMap™ version 10.2) and used ungeneralised (high-resolution) administrative boundary shapefiles for Ireland from the Central Statistics Office.

#### Geographic variation in availability of day care

To compare the availability of day care across geographic areas we divide the estimated number of day care places for people with dementia by the estimated community dwelling dementia population. The estimated number of people with dementia living in the community in each CHO and CHN area is based on national estimates [3]. While these estimates do not take into account local variation in the proportion of people with dementia living in nursing homes, they provide a population base for comparing the availability of day care across geographic areas.

We first compare the variation in availability across the nine CHO areas. We then compare the availability across the CHN areas. While the comparison of the provision of day care services across the CHN areas is complicated by day care centres that are located at the edge of an area, this approach, when visualised on a map, provides a straight-forward method of indicating areas with potentially low availability.

Day care centres are typically accessed by a bus collection service which collects clients from their homes and brings them to the centre. This limits the catchment area from which people can access the service, as overly long journey times are generally not acceptable [7, 25]. Travel time has been shown to be an important determinant of day care utilisation in a number of previous studies [16, 25–28]. To show the areas of CHNs where access is likely to be more difficult, we identified EDs where the centroid (geographic centre) was more than 15 kms along the road network from a day care centre; this represents the outer boundary of acceptable journey times for a bus collection service.

## Results

The survey shows that there are at least 14,193 unique individuals who typically attend day care centres for 1 to 3 days per week. Three-quarters of attendees are categorised as over 65 without dementia; 5% of attendees are identified as being under 65 years of age; and 20% of attendees are identified as over 65 and having dementia (a total of 2805 individuals with dementia). This represents between 8 and 14% of people with dementia in the community [3], though this is likely to be an underestimate of the number of people with dementia attending day care services due to the under diagnosis of dementia in the population generally and incomplete data in the survey.

As described above, we categorised day care centres into four types: dementia-specific centres; centres with dementia-specific days; centres that provided for people with dementia as part of a generic service; and, centres where no dementia places or cases were recorded. Out of a total of 317 centres, 245 centres (77%) provide places for people with dementia to some extent. Overall, these centres provide a total of 5969 weekly places for people with dementia.

There are 58 dementia-specific day care centres across the country. These centres, mostly run by the ASI (80%), account for 46% of the estimated number of dementia places in the survey. A small number of generic centres [16] have dementia-specific days. These centres provide 5% of dementia places. There are 171 centres that cater for people with dementia within a general service. These centres account for 49% places for people with dementia. A substantial minority of centres (72) did not record any dementia places or cases in the survey. Reasons reported in the survey for not taking people with dementia include an unsuitable building or a lack of appropriate staff.

Table 1 shows the estimated weekly dementia places per person with dementia living in the community by CHO area. Nationally, there are 16.7 places per 100 people with dementia living in the community. However, the table shows a high level of variation in the availability of day care for people with dementia across CHO areas. For example, in CHO 1 there are 14.2 weekly places per 100 people with dementia living in the community, compared to 21.3 per 100 people in CHO 9. These figures show the weekly number of places; the number of unique users will be determined by the number of times per week that individuals attend.

**Table 1:** Estimated weekly dementia places per person with dementia living in the community by Community Health Organisation area

	A	B	(B/A)*100
	Estimated Dementia Cases in Community (2016)^1^	Weekly Places for People with Dementia^2^	Weekly Places Per 100 People with Dementia
CHO 1	3414	485	14.2
CHO 2	3950	723	18.4
CHO 3	3065	553	18.0
CHO 4	5498	860	15.6
CHO 5	4107	632	15.5
CHO 6	3410	571	16.5
CHO 7	4086	669	16.6
CHO 8	4101	600	14.5
CHO 9	4106	876	21.3
Total	35,737	5969	16.7

Notes

1. Estimated dementia cases in community based on Pierse, O Shea [3]. Does not include 19,500 people with dementia living in nursing homes.

2. Weekly places for people with dementia in all centre types.

**Fig. 1:**
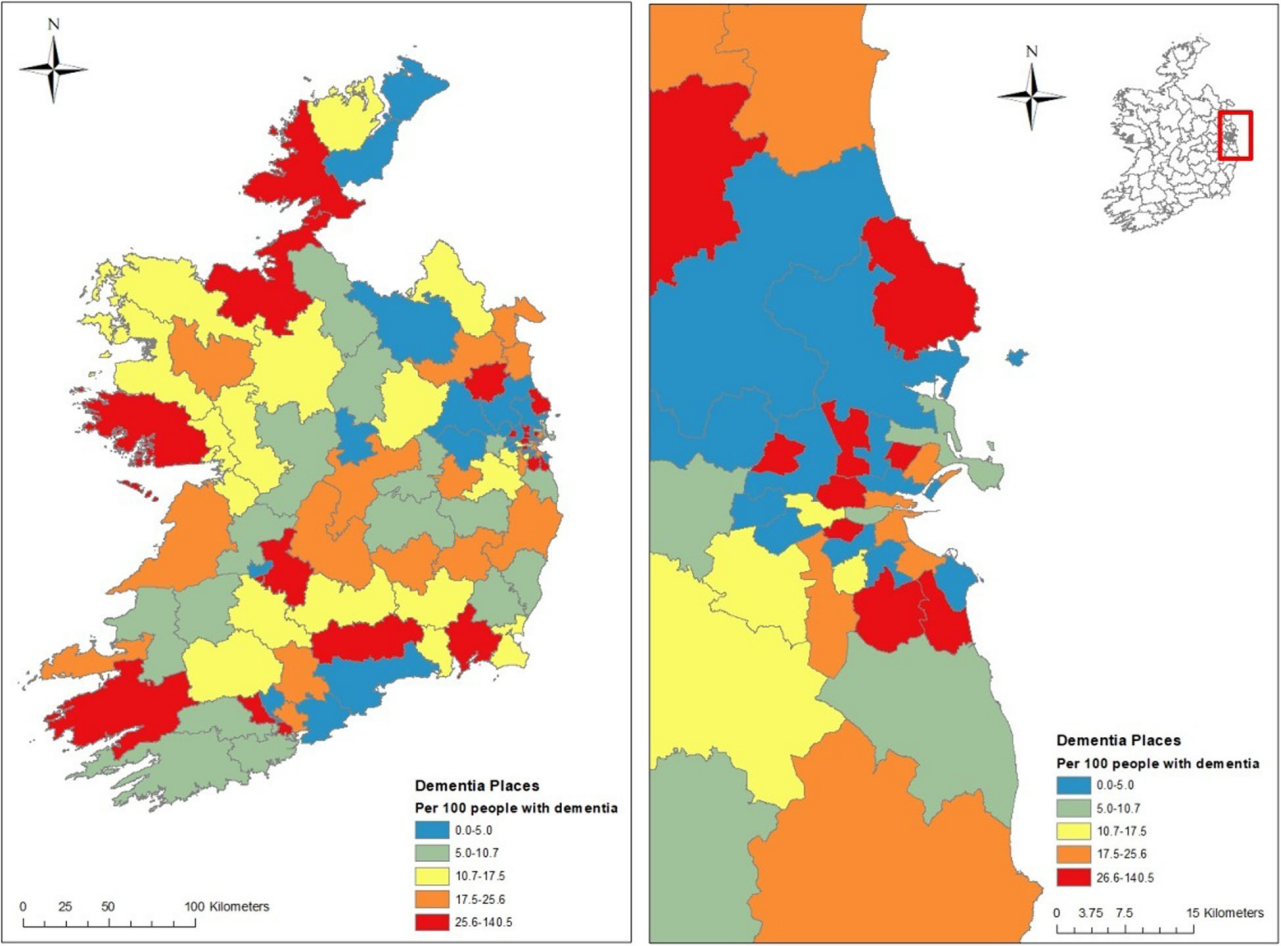
Dementia day care availability by Community Health Network area for Ireland (left panel) and Dublin (right panel)

Within each of these CHO areas, day care services that cater for people with dementia are not evenly distributed across CHN areas. Figure 1 shows the geographic variation across CHNs in the number of weekly dementia places per 100 persons with dementia in the community for Ireland and the Dublin region. Specifically, the maps show the quintiles (5 equal groups) of the 96 CHN areas based on the number of dementia places. So, for example, the areas in the bottom quintile (shown in blue) have a day care availability of fewer than 5.0 weekly places per 100 people with dementia in the community, while the areas in the top quintile (shown in red) have more than 25.6 weekly places per 100. Some of the areas with low availability, particularly in urban areas, are adjoined by areas with very high availability, though many are not. This approach shows the parts of the country that have more places than others.

**Fig. 2:**
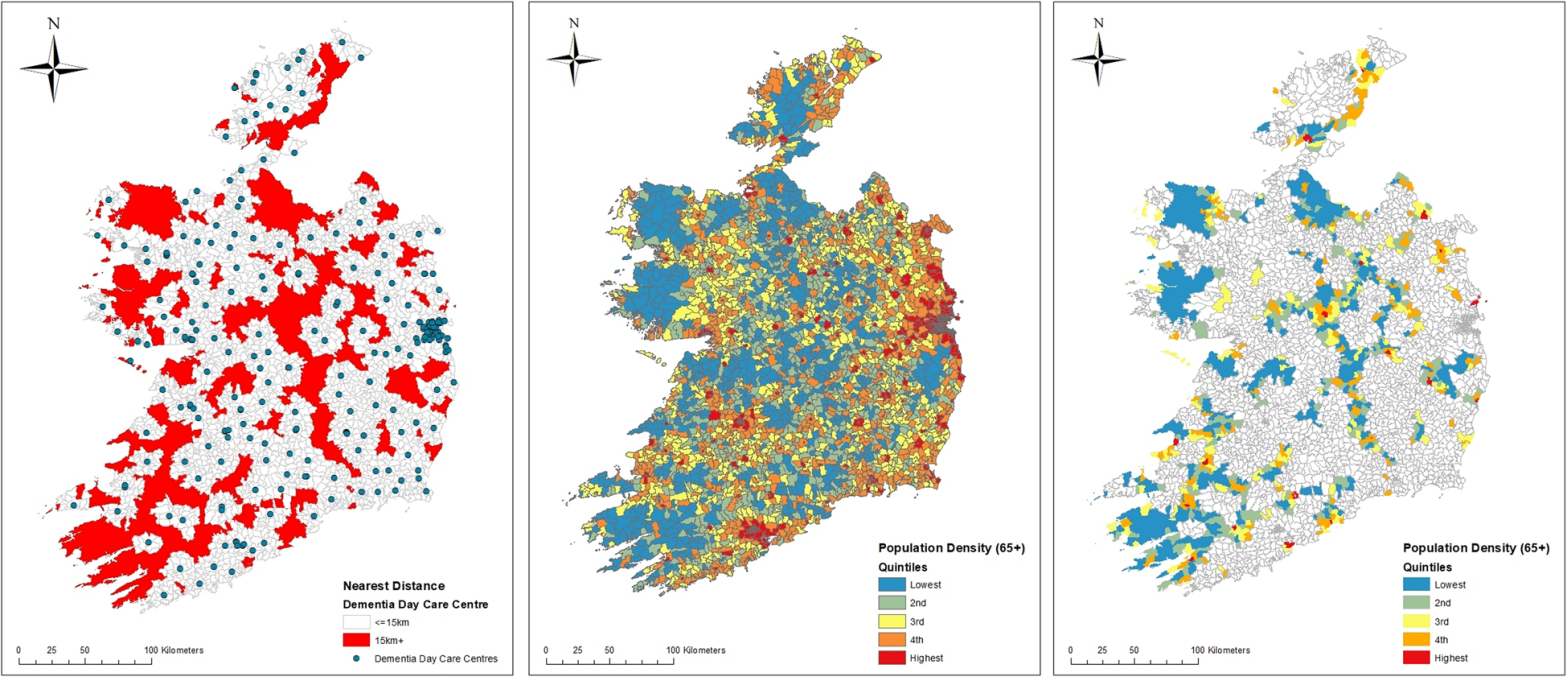
Accessibility and population density. Distance to dementia day care centres; areas with poor accessibility in red (left panel). Quintiles of number of people over 65 years per square km (middle panel). Number of people over 65 years per square km (quintiles) in areas of poor accessibility (right panel)

In addition to availability, we also look at the geographic accessibility of day care services for people with dementia, in particular the distance people with dementia live from a centre. This is particularly important in rural areas where service locations are further apart. Figure 2 (left) shows the EDs (in red) whose centre is more than 15kms from a day care centre that caters for people with dementia. Overall, an estimated 18% of people with dementia live in these areas, which are likely to be beyond the range of a day care bus service. Overall the map shows that while most areas of the country are within the catchment area of a day care centre that caters for people with dementia, there are substantial parts of the country where geographic accessibility is likely to be a significant issue.

While Fig. 2 (left) provides a good indication of geographic accessibility to day care centres, it is also important to consider population density, since it could be the case that areas with poor accessibility also have relatively low potential need for a service. Thus, Fig. 2 (middle) presents information, again by ED, on the numbers of people aged over 65 years per square km. Figure 2 (right) then combines both maps to highlight the population density in the areas with poor accessibility. It shows, for example, areas in blue and green that have poor access to services but also have low population densities. On the other hand, areas in red and orange have poor accessibility and relatively high population densities. The majority of EDs with poorer accessibility to day care services for people with dementia also have low population densities. However, it is the areas with poor accessibility and high population densities that may be of most interest to government.

### Illustrative example: the local allocation of investment

**Fig. 3:**
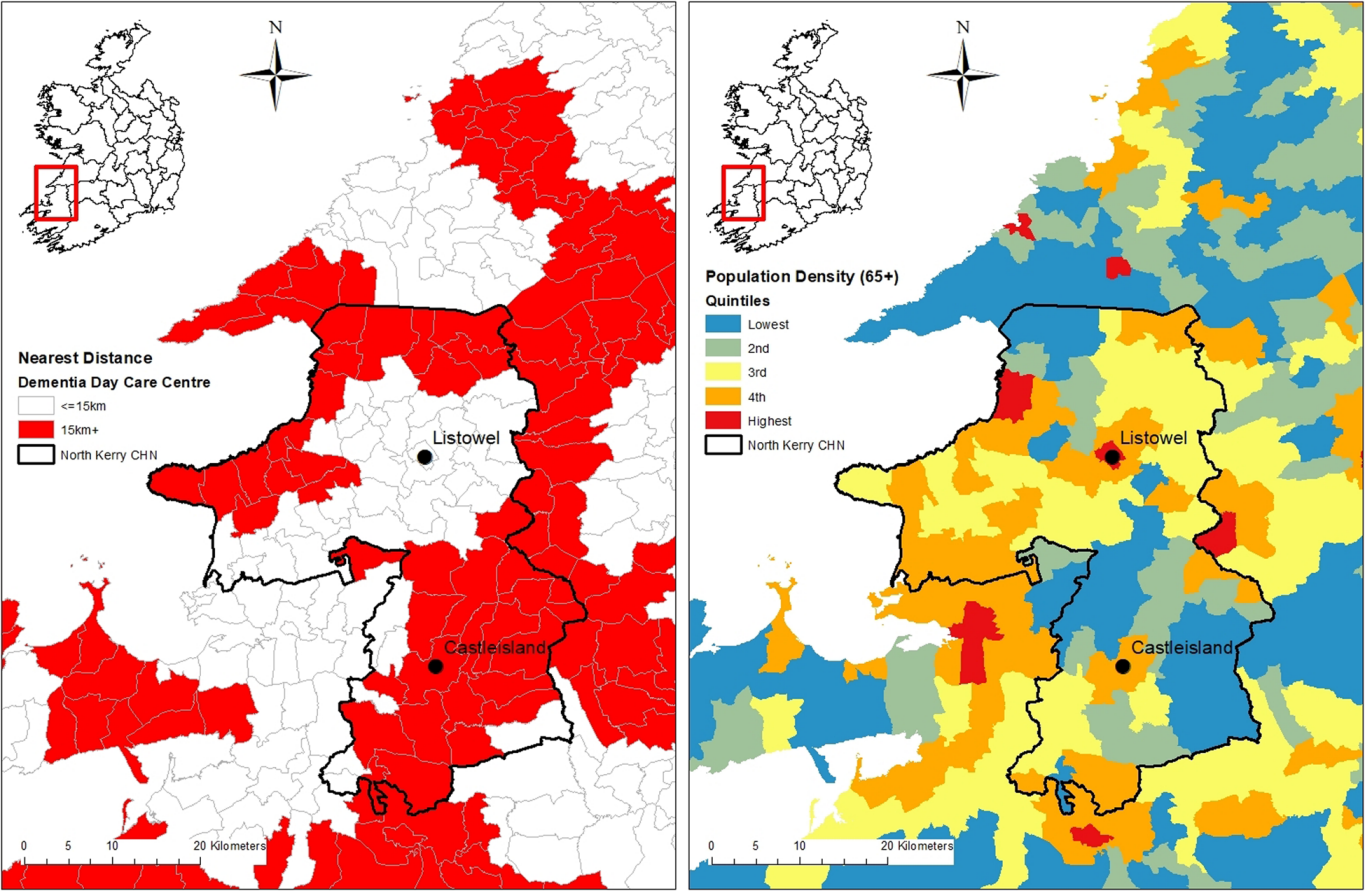
Illustrative example: North Kerry Community Health Network. Distance to dementia day care centres. Areas with poor accessibility in red (left panel). Quintiles of number of people over 65 years per square km (right panel)

To provide an example of how this approach can be used at a local level for service planning, we look at a hypothetical decision to develop the provision of day care for people with dementia in one CHN area, North Kerry, shown by the black boundary in Fig. 3. At present, day care provision exists in two of the main towns, Listowel and Castleisland. Listowel has 75 generic weekly day care places and 30 dementia-specific day care places, while Castleisland has 140 generic weekly day care places but no dementia-specific service. So what can a GIS analysis tell us about where any potential new day care centre should be located? Although the area surrounding Listowel has relatively good geographic accessibility to dementia day care services (Fig. 3 left), it has comparatively high population density and potential demand; there are an estimated 167 people with dementia in the 15 km catchment area (Fig. 3 right) based on prevalence data estimated using the 2016 CSO census of the Irish Population and the international literature on prevalence [29]. In contrast, the area surrounding Castleisland has poor accessibility to dementia day care (Fig. 3a) but has comparatively lower population density and lower demand than Listowel, with approximately 89 people with dementia in the 15 km catchment area (Fig. 3 right). Thus, this information, derived from a GIS analysis such as presented in Fig. 3, can provide valuable information to aid planning decisions linking existing availability, population density and dementia prevalence. However, it is important to stress that other factors may also influence the final decision on the location of new day care centres. For example, day care centres in neighbouring CHNs may provide accessible places for people on the outskirts of this CHN. In addition, the availability of other services, for example a well-functioning Alzheimer café in either of the towns, may mean that demand for day care is not as high as the other town. Nonetheless, the GIS information provides vital objective information which can contribute to the decision.

## Discussion

This paper uses GIS methods to provide evidence for policy decisions relating to the allocation of resources for day care provision in Ireland. A significant investment in day care services is required to maintain or increase the current service for people with dementia, due to the increasing number of people living in the community with dementia. The estimated average growth rate in the number of people with dementia in Ireland is 3.6% per year to 2030 [4]. Thus, just to maintain the current level of service, an estimated 4433 new weekly dementia day care places will be required by 2031. Prior to this research it has not been possible to identify variation in the availability and accessibility of day care services for people with dementia across the country. Therefore, our GIS approach provides valuable evidence that can help inform decisions relating to resource allocation and service provision in this sector in the future.

In some cases, increased capacity could be achieved by increasing the number of days that current facilities are providing. In other cases, where buildings are unsuitable or the current facilities are at maximum capacity, new facilities will be required. An important issue to be considered is whether additional capacity should be provided through dementia-specific centres, dementia-specific days in generic centres, or through a good quality generic day care service. Unfortunately, there is little evidence on which of these is the best model of delivery [30, 31]. A number of respondents to the survey commented that it was better to incorporate people with dementia into a general service and this may offer more scope for enhanced provision, particularly in areas with low population densities. However, the inclusion of people with dementia in a general day care service will require action in relation to staff training, staffing levels and the development of the physical environment.

In terms of resource allocation decisions, public resources in Ireland are currently allocated for day care services for people with dementia through block grants to the Alzheimer Societies and through funding for individual centres from the Older Persons Services Budget in each CHO area. In this paper we take a multi-level approach to the resource allocation problem of identifying the parts of the country with the greatest need for day care services for people with dementia, taking account of existing provision, population density, dementia prevalence and accessibility, measured by distance. The GIS methods we employ provide a practical and easily replicated decision making framework for the allocation of regional and sub-regional budgets, and for informing the locations where new services may be required.

For planning at a national and regional level, the main focus of the resource allocation framework we outline is towards balancing the availability of day care services for people with dementia across CHO and CHN areas. For planning at a local level, both population density and accessibility are key considerations. By providing an illustrative example, we have shown how a decision about where to locate increased capacity for day care services for people with dementia could be approached. The population density map shows parts of a CHN area where it may be most beneficial to provide a day care service in terms of potential demand. The accessibility map shows parts of a CHN area that have poor accessibility to dementia day care facilities and for these areas, alternative solutions may also be required. For example, it may be cost effective to use a generic day care centre in that area, to provide for people’s needs in their own home, or to put increased resources into other transport options, such as volunteer drivers.

In terms of strengths and limitations, this study benefits from the availability of a comprehensive national survey of day care services. However, because of the low rates of dementia diagnoses, and the poor recording of dementia diagnoses, there may be an under-estimation of the numbers of clients with dementia and hence the number of existing day care places for people with dementia. In addition, due to the limitations of the data collection infrastructure in day care centres, a significant level of data cleaning and processing was required to estimate the number of places utilised by people with dementia in each day care centre. These factors may have resulted in over/under estimates of the number of dementia places in some centres. In addition, this study does not examine issues such as utilisation rates and waiting times which are likely to be influenced by a broad range of factors such as the quality of the service and perceptions of day care. Nonetheless, the comprehensiveness of the survey data allowed us to generate a range of data and maps which, when used collectively, show the variation in day service provision across the country. At the very least, the maps can be an important aid to the resource allocation decision-making process.

## Conclusions

This paper provides a GIS based approach that can help inform future resource allocation decisions for day care services for people with dementia at regional, sub-regional and local levels. The mapping of the availability of day care services for people with dementia shows substantial geographic variation across the country. In addition, there are large parts of the country where day care services are difficult to access. However, many of these areas have a low population density. These maps can be used to assist in targeting investment to areas where need is greatest.

## Data Availability

The data that support the findings of this study were provided by the Health Service Executive and are not currently publicly available. Data are however available from the authors upon reasonable request and with permission of the Health Service Executive.

## Abbreviations

HSE: Health Service Executive
GIS: Geographic Information Systems
CHO: Community Health Organisations
CHN: Community Health Network
ED: Electoral division
CSO: Central Statistics Office
ASI: Alzheimer Society of Ireland
